# Regulation of Mitochondrial Function by Natural Products for the Treatment of Metabolic Associated Fatty Liver Disease

**DOI:** 10.1155/2021/5527315

**Published:** 2021-06-16

**Authors:** Tingting Shi, Liping Yu, Rangxiao Zhuang, Jianjun Xi, Ruoyu He, Yidan Shao, Jinsong Huang, Shourong Liu, Xingxin Yang

**Affiliations:** ^1^The Hangzhou Xixi Hospital Affiliated to Zhejiang Medical University, Hangzhou 310023, Zhejiang, China; ^2^College of Pharmaceutical Science, Yunnan University of Traditional Chinese Medicine, 1076 Yuhua Road, Kunming 650500, Yunnan, China

## Abstract

Metabolic associated fatty liver disease (MAFLD) is a multifactorial systemic disorder that occurs in the absence of excessive alcohol consumption. The disease is characterized by fatty degeneration and fat accumulation in liver parenchymal cells, the incidence of which is increasing annually, particularly in younger adults. MAFLD is caused by genetic and metabolism related disorders, of which mitochondrial dysfunction is the major contributor. Natural products can relieve MAFLD through restoring mitochondrial function. In this article, we describe the relationship between mitochondria and MAFLD and discuss the beneficial effects of natural products as a future anti-MAFLD strategy. *Significance Statement*. We herein propose that the development of mitochondrial regulators/nutrients from natural products can remedy mitochondrial dysfunction which represents an attractive strategy for the treatment of MAFLD. Furthermore, the mitochondrial regulation of natural products can provide new insight into the underlying mechanisms of action of natural products used for future MAFLD therapeutics.

## 1. Introduction

Metabolic associated fatty liver disease (MAFLD) is a metabolic stress-related liver injury that is closely associated with insulin resistance and genetic susceptibility. The spectra of the disease include nonalcoholic liver steatosis, nonalcoholic steatohepatitis, cirrhosis, and hepatocellular carcinoma. MAFLD can lead to disability and mortality and is closely related to the metabolic syndrome, type 2 diabetes, arteriosclerotic cardiovascular disease, and colorectal tumors [1]. With changes in lifestyle and the control of viral liver disease, the incidence of MAFLD is increasing, with a prevalence of ∼25% in adults worldwide. To date, MAFLD has emerged as the most common chronic liver disease (49.3%) in China [2, 3]. Despite this, anti-MAFLD therapeutics are lacking and new and more effective therapies through an increased understanding of disease pathogenesis are urgently required.

Mitochondria are the major sites of oxidative metabolism in which carbohydrates, fats, and amino acids are oxidized to release energy. Fatty acid *β*-oxidation occurs primarily in the mitochondria, which, when impaired, leads to fat accumulation in the liver, ROS production, and hepatocyte apoptosis [4]. As changes in mitochondrial function often precede the occurrence of clinical symptoms, the timely detection of its functional changes and the implementation of appropriate therapeutics can prevent or delay the occurrence and development of MAFLD.

It has been reported that an array of natural products [4] can alleviate MAFLD-related symptoms through the regulation of mitochondrial function. The aim of this review was to examine current knowledge of the relationship between mitochondria and MAFLD, and the regulation of natural products to the mitochondria for the treatment of MAFLD. We herein propose that the development of mitochondrial regulators/nutrients from natural products can remedy mitochondrial dysfunction which represents an attractive strategy for the treatment of MAFLD. Furthermore, the mitochondrial regulation of natural products can provide new insight into the underlying mechanism (s) of action of natural products used for future MAFLD therapeutics.

## 2. Role of Mitochondria in the Occurrence and Development of MAFLD

### 2.1. Energy Metabolism Disorders

The three major nutrients in organisms, namely, carbohydrates, fats, and proteins, all undergo oxidative phosphorylation in the mitochondria through the tricarboxylic acid cycle to generate energy. MAFLD occurs in response to energy metabolism disorders and is therefore intricately linked to mitochondrial function.

Mitochondria provide more than ∼90% of the energy within cells [5]. Mitochondrial function in healthy cells is directly related to whole body energy metabolism. Accordingly, disorders in mitochondrial function and their reserves in MAFLD patients' lead to the accumulation of fat in the liver and subsequent liver injury. Mitochondrial damage leads to dysfunction in the electron transport chain, altered mitochondrial protein expression, oxidative phosphorylation, and mitochondrial DNA damage. These changes culminate in a loss of ATP synthesis which impairs the growth and metabolism of hepatocytes. When hepatocytes fail to function correctly, apoptosis frequently occurs [4].

### 2.2. Mitochondrial DNA Damage

Mitochondrial deoxyribonucleic acid (mtDNA) is the only genetic material found in an organelle outside the nucleus. MtDNA plays a crucial role in oxidative phosphorylation and MAFLD. When cellular damage or cell stress occur, mtDNA is released from the mitochondria and is considered an important regulatory molecule of innate immune responses, which can induce the occurrence of inflammatory disease [6]. During the formation of MAFLD, continuous inflammation leads to tissue damage and the overproduction of ROS. An important factor in inflammatory response amplification in MAFLD injury is the activation of endogenous “danger signals,” namely, DAMPs (mainly including mtDNA). Damaged tissues and cells release mtDNA which can activate the inflammatory response, subsequently inducing liver damage.

Mitochondria produce adenosine triphosphate (ATP) through oxidative phosphorylation. Reactive oxygen species (ROS) are by-products of the respiratory chain, meaning mitochondria are the major cellular source of ROS. Studies have indicated that the MAFLD-related injury induced by mtDNA damage can affect the respiratory chain, enhance oxidative stress and inflammatory responses, and induce apoptosis [7]. In addition, mtDNA damage can inhibit the production of ATP in the mitochondria, leading to cell dysfunction and subsequent tissue damage [7].

Due to the lack of histone protection and complete mutational repair functions, the mutation rates of mtDNA are high. MtDNA is easily attacked by intracellular ROS, leading to base pair deletions and mutations which further disturb lipid metabolism in hepatocytes. Studies by Kamfar et al. [8] revealed that the copy number of mtDNA in hepatocytes is key to the susceptibility to MAFLD.

### 2.3. Oxidative Stress and Lipid Peroxidation

Oxidative stress and lipid peroxidation are the major causative factors of MAFLD. Oxidative stress is a pathological state that occurs in response to the generation of free radicals or ROS by oxygen molecules, which exceeds their detoxification ability. Upsetting the dynamic balance between oxidants and antioxidants promotes oxidative stress in the mitochondria. In patients with MAFLD, mitochondrial function declines, leading to a loss of ATP synthesis, decreased ROS consumption, and ROS accumulation. This leads to lipid deposition in the liver and increased oxidative stress responses, ultimately leading to hepatocyte apoptosis.

Oxidative stress and mitochondrial dysfunction occur in patients with MAFLD [9]. Mitochondrial dysfunction induced liver steatosis leads to excessive ROS levels, oxidative stress, and lipid peroxidation, ultimately disrupting the mitochondrial respiratory chain [10] and energy metabolism, as a result of mitochondrial damage [11].

Lipid peroxidation is a process in which ROS oxidizes biological membranes in situations of elevated oxidative stress. ROS reacts with macromolecular substances including polyunsaturated fatty acid side chains to generate lipid peroxides that increase endogenous ROS levels. In addition, lipid peroxidation products lead to the loss of mtDNA, replication errors, and the inhibition of mtDNA repair in patients with MAFLD, thereby reducing the activity of the respiratory chain complex. Lipid peroxides can combine with mitochondrial proteins to form adducts that inhibit electron transfer in the respiratory chain. The increased free fatty acids in the liver can induce oxidative stress responses, leading to reduced mitochondrial function and aggravated MAFLD.

Furthermore, excessive ROS production in the mitochondria can oxidize macromolecular substances, leading to further oxidation and reoxidation damage to both proteins and lipids. As a result, ROS induces a series of pathological changes that ultimately lead to liver damage.

### 2.4. Hepatocyte Apoptosis

Hepatocytes undergo apoptosis through death receptor pathways and mitochondrial-dependent apoptotic pathways. Mitochondria are the regulatory centers of cell apoptosis. Apoptosis-related factors such as cytochrome C (Cytc) and apoptosis-inducing factor (AIF) in the mitochondria are released into the cytoplasm in response to changes in the mitochondrial membrane potential, leading to the activation of downstream caspases and apoptosis [12].

The change in mitochondrial membrane permeability is an important aspect of the occurrence of apoptosis and necrosis. Membrane permeability is mainly affected by the regulation of mPTP on the inner mitochondrial membrane. Cells survive only when mitochondrial pores are closed, as their excessive opening leads to apoptosis. Kang et al. [13] found that the apoptotic rates of hepatocytes in MAFLD rats fed with a high-fat diet increased when the mPTPs were open. This led to an increase in membrane permeability, a loss of mitochondrial membrane potential, the release of apoptosis-inducing factors, and the subsequent activation of proapoptotic proteins, ultimately leading to apoptosis. Xiao et al. [14] reported that ginsenoside Rg1 could inhibit hepatocyte apoptosis in MAFLD rat models and alleviate disease progression. Cai et al. [15] found that the saponins of *Gynostemma* could effectively inhibit oxidative stress responses in the hepatocytes of MAFLD rats, thereby displaying hepatoprotective effects.

It can therefore be concluded that the mitochondrial damage caused by MAFLD is closely related to the induction of apoptosis in liver cells. With a decrease in mitochondrial membrane potential, Cytc is released from the mitochondrial membrane into the cytoplasm, leading to caspase activation and hepatocyte apoptosis.

### 2.5. Mitophagy

Mitophagy is a selective form of autophagy that eliminates dysfunctional mitochondria. The regulation of mitophagy can be either ubiquitin-dependent or non-ubiquitin-dependent. Ubiquitin-dependent mitophagy includes PTEN-induced kinase 1 (PINK1)/E3 ubiquitin ligase parkin-mediated mitophagy and parkin-independent mitophagy. Ubiquitin-independent mitophagy refers to the mitophagy mediated by mitochondrial autophagy receptors. Damaged mitochondria can be removed by mitophagy to avoid the toxic effects of ROS on cells. The damage/death of hepatocytes occurs as a result of dysregulated mitophagy, highlighting its role in cellular homeostasis.

A variety of liver-related diseases (including MAFLD) are related to mitophagy [16–20]. Lipid autophagy can selectively recognize and degrade lipids, thereby maintaining lipid homeostasis in hepatocytes [19]. Adipogenic autophagy is an important mechanism through which cells regulate lipid balance in the liver and is key to cell metabolism and organelle renewal. Mitophagy regulates mitochondrial quality to maintain cell homeostasis [20].

Koga et al. [21] found that changes in the membrane structure of autophagosomes occurred as a result of lipid deposition, which in turn affected their fusion with lysosomes, leading to reduced lipid degradation by autophagy during the early stages of MAFLD. When autophagy induction fails to remove cellular inflammatory factors, damaged organelles and excessive ROS, liver tissue injury, liver cell edema, liver tissue necrosis, and inflammatory cell infiltration, resulting in NASH, occur [22]. Autophagy is closely related to MAFLD [23] and represents a therapeutic target for the prevention and treatment of liver failure.

### 2.6. Fatty Acid Metabolism

Fat metabolism mainly occurs in the liver. When fat synthesis and decomposition are imbalanced, or the output is obstructed, fat excessively accumulates, leading to the development of fatty liver [24]. The main form of fatty acid oxidation is *β*-oxidation, which mainly occurs in the mitochondria and plays a key role in fatty acid metabolism [25]. Lipid metabolism disorders lead to increases in free fatty acids, disorders of liver fat metabolism, and increased TG synthesis in liver cells, resulting in excessive lipid accumulation. Excessive TG and NEFA accumulate in hepatocytes and are oxidized in the mitochondria, which produces excessive ROS and triggers inflammatory reactions, causing further damage to the liver tissue.

Lipid metabolism is regulated by the expression of enzymes and genes related to fatty acid metabolism [26, 27]. Amongst them, PPAR and its downstream target genes in the liver tissue increase fatty acid oxidation and inhibit fatty acid synthesis, thus improving abnormal fatty acid metabolism and blood lipid levels. This has been proposed as a therapeutic strategy to treat pathological obesity and nonalcoholic fatty liver [28]. In obese subjects, type 2 diabetes and insulin resistance are common. In such cases, the sources of fatty acids in liver cells increase, as does the oxidation output, resulting in the deposition of TG in the liver, increasing the risk of NAFLD development [29].

### 2.7. Morphological Changes

The mitochondrial cristae of normal hepatocytes form clear and abundant matrix particles of high electron density [30–32]. The liver pathology of MAFLD is characterized by a dense distribution of fat in the mitochondria, obvious mitochondrial swelling, the shortening of cristae, and rupture of the external membranes.

## 3. Effects of Natural Products on MAFLD

MAFLD is a metabolic syndrome that induces a series of pathological changes including alterations in glucose and lipid metabolism and mitochondrial function. Studies have shown that many natural products (including mixtures and monomers) alleviate MAFLD through their regulation of mitochondrial function (Table 1). Their main functions include improving energy metabolism, the protection of mtDNA, alleviating oxidative stress and lipid peroxidation, inhibiting hepatocyte apoptosis, regulating mitophagy, promoting fatty acid metabolism, and improving mitochondrial morphology in hepatocytes.

Additionally, patients with coeliac disease (CD) have to follow a lifelong gluten-free diet (GFD) [85]. However, GFD is related to increased lipid and carbohydrate intake [86–93]. Thus, many patients with CD become overweight after GFD treatment [85]. About 3% of patients diagnosed with MAFLD actually have an underlying CD. Natural products may be used to remedy GFD-indued MAFLD that merit further investigation.

## 4. Summary

**Figure 1:**
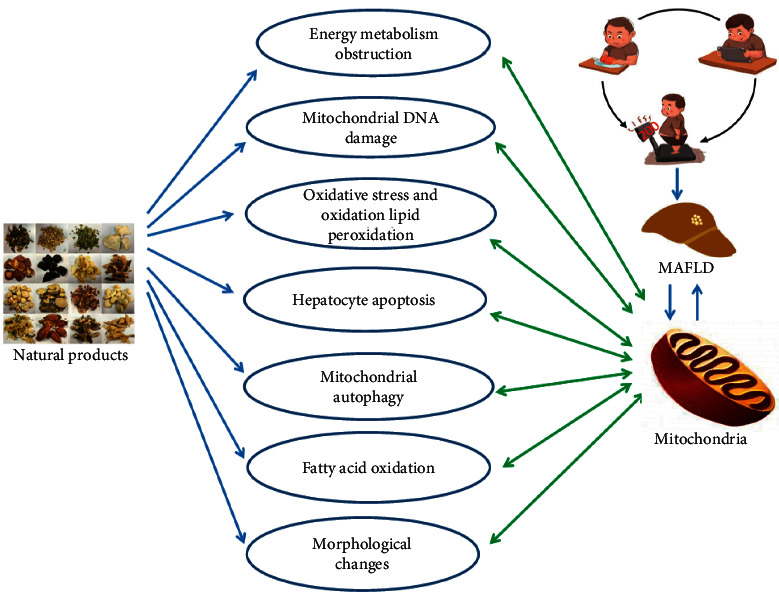
Protecting the mitochondria to cure MAFLD using natural products. MAFLD: metabolic associated fatty liver disease.

MAFLD shows the pathological characteristics of excessive mitochondrial damage due to the weakened clearance of dysfunctional mitochondria. Natural products can regulate mitochondria to alleviate MAFLD states (Figure 1). However, the active ingredients of many natural extracts and their specific interactions with mitochondrial proteins remain largely undefined. Further in-depth studies on the regulation of mitochondria by natural products are now required to define the mechanisms of MAFLD resistance and to improve drug development and the subsequent clinical treatment of MAFLD. It is believed that the increased discovery of natural products that can remedy mitochondrial dysfunction have the potential for the treatment of MAFLD.

## Figures and Tables

**Table 1:** Regulating mitochondria to prevent MAFLD by natural products.

Type of natural product	Natural product	Mitochondrial regulation	Experimental models
Mixture	Tangshen prescription [33]	Restoration of autophagy in damaged fatty liver and reduced mitochondrial damage caused by ROS	MAFLD mouse models induced by a high-fat or choline-methionine-deficient diet
	Zhifang prescription [34]	Increased expression of Mfn1 and Opa1, which promote mitochondrial fusion and enhance mitochondrial autophagy	MALFD rat models induced by a high-fat diet
	Yinchen Linggui Zhugan decoction [35]	Activation of autophagy, balancing the body's oxidation and antioxidation systems, improving NASH	MALFD rat models induced by a high-fat diet
	Tiaogan lipi prescription [36]	Improves MAFLD by increasing autophagy levels	MALFD rat models induced by a high-fat diet
	Baohe pills and Baohe pills added with Polygoni Cuspidati Rhizoma et Radix [37]	Reduced mitochondrial swelling, increasing the number of mitochondria, and maintaining mitochondrial function and integrity	MAFLD rat models induced by modified high-fat emulsion
	Sini San [38]	Ability to resist lipid peroxidation, increase ATPase activity, reduce mitochondrial swelling, and increase mitochondrial membrane potential	MAFLD mouse models induced by methionine choline deficiency
	Erchen decoction [39]	Increased ATP synthesis and restoration of mitochondrial energy metabolism disorders	MAFLD mouse models induced by a high-fat diet
	Shuganjianpi Huatanhuoxue prescription [40]	Reduced lipid peroxidation, accelerated *β*-oxidation in the mitochondria	MAFLD in vitro cell models
	Fufang Zhajin granules [31]	Improves mitochondrial lipid metabolism in liver cells	MALFD rat models induced by a high-fat diet
	Huatan Qushi Huoxue prescription [41]	Increased number of mitochondria and their cristae, enhanced liver cell energy metabolism, and restoration of mitochondrial morphology and function	NASH rat models induced by a high-fat diet combined with tetracycline intraperitoneal injection
	Ganshu decoction [42]	Reduced mitochondrial swelling, improved mitochondrial membrane fluidity, and regulation of mitochondrial lipid oxidation in liver cells	MALFD rat models induced by a high-fat diet
	Ganshule tablets [43]	Increased mitochondrial fatty acid *β*-oxidation and higher number of mitochondrial cristae	MALFD rat models induced by a high-fat diet
	Ganzhikang capsules [44]	Decreased synthesis of NEFA and TG, enhanced liver function and oxidation of fatty acids, and ability to scavenge free radicals and the products of lipid peroxidation	MALFD rat models induced by a high-fat diet
	Jiawei Zhaqu decoction [45]	Improves lipid metabolism in the mitochondria, reduced UCP-2 and COX I production	MALFD rat models induced by a high-fat diet
	Jianpi Shugan Jiangzhi prescription [46]	Increased number of mitochondria and cristae, enhances ATP synthesis and energy metabolism, and increases fatty acid metabolism	MAFLD mouse models induced by a high-fat diet and 10% CCL_4_ edible oil solution
	Qingzhi Hugan prescription [47]	Reduced mitochondrial swelling and improved mitochondrial morphology	MALFD rat models induced by a high-fat diet
	Tiaogan Quzhi prescription [48]	Reduced mitochondrial swelling, and improved mitochondrial morphology	MAFLD rat models induced by a high-fat diet
	Xiaoyu Huatan decoction [49]	Reduces mitochondrial swelling, increased number of mitochondria, increased ATP synthesis and mitochondrial energy reserves, and increased fatty acid metabolism	MALFD rat models induced by a high-fat diet
	Yishen Tiaogan prescription [50]	Increases the number of mitochondria and the stability of membrane potential and improves the activity of cytochrome oxidase and the self-repair processes of damaged mitochondrial DNA	MALFD rat models induced by high-fat diets
	Zhigan prescription [4,51]	Ability to reduce mitochondrial energy metabolism disorders, mitochondrial swelling in liver tissues, and ability to regulate mitochondrial autophagy	MALFD rat models induced by a high-fat diet
	Shiwei Ganzhikang capsules [52]	Protection and repair of the mitochondrial membranes of liver cells and ability to promote the recovery of liver cell functions	MALFD rat models induced by high-fat diets
	Allium Fistulosum bulbus [53,54]	Improves mitochondrial respiratory function, increases mitochondrial biosynthesis, and promotes fatty acid oxidation	MALFD rat models induced by a high-fat diet
	Blueberry [55]	Reduction of lipid peroxides, regulation of energy metabolism in hepatocyte mitochondria, maintenance of the balance between oxidation and antioxidation, and reduced oxidative stress responses in the liver	MALFD rat models induced by a high-fat diet
	Sibiraea angustata [56]	Strengthen *β*-oxidation of fatty acids in the mitochondria	MALFD rat models induced by a high-fat diet
	Granati Pericarpium [57]	Enhanced antioxidant capacity and maintenance of stable mitochondrial functions	MALFD rat models induced by a high-fat diet
	Sida orientalis [58]	Improves mitochondrial oxidative stress	—
	Gecko [59]	Ability to resist lipid peroxidation, prevents oxidative stress, reduces the production of lipid peroxides, and prevents cell apoptosis	MAFLD mouse models induced by a high-fat diet
	*Trillium tschonoskii* [60]	Reduces mitochondrial swelling	MAFLD rat models induced by a high-fat diet combined with the intraperitoneal injection of carbon tetrachloride solution
	*Gynostemma pentaphyllum* [61]	Ability to adjust the molecular structure of mitochondrial cardiolipin and improved mitochondrial functions	Primary hepatocytes cultured in high glucose
	Extract of Polygoni Multiflori Radix [62]	Prevents the *β*-oxidation of mitochondrial fatty acids and improves liver lipid metabolism	MAFLD mouse model induced by an MCD diet
	*Rhodiola crenulata* extract [63]	Improves insulin resistance, downregulates lipid synthesis in the liver	MAFLD models of C57BL/6 mice induced by a high-fat diet
	Polysaccharides of *Cordyceps* [64]	Reduces mitochondrial swelling and increases the number of mitochondrial cristae	MAFLD rat models induced by a high-fat emulsion
	Total flavonoids of *Litsea Coreana* [65]	Increases the number of mitochondrial cristae, improves mitochondrial morphology and function	MAFLD rat models induced by a fat emulsion gavage
	Notoginseng total saponins [66]	Decreases hydroxyl free radicals in the mitochondria of liver cells, reduces MDA concentrations, and increases total superoxide dismutase activity and the total antioxidant capacity of serum	—
	Polysaccharides of *Ganoderma lucidum* [67]	Improves mitochondrial ultrastructure, reduces mitochondrial swelling, lowers cytochrome C levels, reduces the activity of apoptotic proteins, and increases mitochondrial oxidation and related enzyme activities	MALFD rat models induced by a high-fat diet
	Pomegranate polyphenols [68]	Increases ATP content, inhibits mitochondrial protein oxidation, and improves the activity of mitochondrial complex enzymes in the liver	MALFD rat models induced by a high-fat diet
Monomer	Hesperidin [69]	Reduces mitochondrial swelling and increases the number of mitochondrial cristae	MAFLD rat models induced by a fat emulsion gavage and sucrose feeding
	Dihydromyricetin [70]	Regulates the SIRT3 pathway to promote the expression of mitochondrial DNA coding genes, maintains the enzymatic activity of the mitochondrial respiratory chain complex, and increases mitochondrial ROS scavenging activity	MALFD rat models induced by a high-fat diet
	Polydatin [71]	Enhances the body's antioxidant capacity, reduces the production of lipid peroxides, and improves the *β*-oxidation of mitochondrial fatty acids	MALFD rat models induced by a high-fat diet
	Salvianolic acid [72]	Protects mitochondria, regulates lipid metabolism, controls oxidative stress and lipid peroxidation, and inhibits apoptosis	MALFD rat models induced by a high-fat diet
	Baicalin [73]	Inhibits the formation of mitochondrial ROS, increases mitochondrial ATP synthesis, and restores the activity of respiratory chain complexes I and II	MAFLD rat models induced by a methionine choline-deficient diet
	Betaine [74]	Its effect of reducing lipid accumulation is achieved by inhibiting the expression of obesity-related genes and N6-methyladenosine demethylation, thereby improving mitochondrial functions	—
	Curcumin [75–77]	Attenuates oxidative stress and the expression of inflammatory factors, alleviates steatosis in MAFLD rats through the activation of autophagy and the prevention of mitochondrial apoptosis	MAFLD rat models induced by high-sugar and high-fat diets
	Quercetin [78]	Improves mitochondrial morphological damage and dysfunction in the liver, promotes mitochondrial biosynthesis, promotes mitochondrial fusion and division, enhances PINK1-parkin-mediated mitochondrial autophagy levels, and improves mitochondrial homeostasis	MAFLD models of C57BL/6 mice induced by a high-fat diet
	Rhein [79]	Reduces mitochondrial swelling and deformation	MALFD rat models induced by a high-fat diet
	Sophocarpine [80]	Inhibits the synthesis of inflammatory cytokines, downregulates UCP-2, and increases the rate of mitochondrial lipid oxidation	MALFD rat models induced by a high-fat diet
	*α*-Mangostin [81]	Reduces the activity of apoptotic proteins, increases mitochondrial oxidation rates and related enzyme activities	MALFD rat models induced by a high-fat diet
	Oxymatrine [82]	Increases CPT-1 enzyme activity and the *β*-oxidation of fatty acids in the mitochondria	MAFLD rat models induced by a high-fructose diet
	Sennoside A [83]	Protects mitochondrial structure and function by targeting VDAC1	MAFLD mice models induced by a high-fructose diet
	Resveratrol [84]	Increases the number of mitochondria	MAFLD rat models induced by a high-fructose diet

ATP, adenosine triphosphate; COX I, cytochrome oxidase I; CPT-1, carnitine acyl transferase-1; DNA, deoxyribonucleic acid; MAFLD, metabolic associated fatty liver disease; MDA, malondialdehyde; Mfn1, mitofusin1; NASH, nonalcoholic steatohepatitis; NEFA, nonesterified fatty acid; Opa1, optic atrophy proteins; ROS, reactive oxygen species; TG, triglyceride; UCP-2, mitochondrial uncoupling protein 2; VDAC1, recombinant voltage-dependent anion channel protein 1; SIRT3, sirtuin-3.

## Data Availability

No data were used to support this study.

## Abbreviations

AIF: Apoptosis-inducing factor
ATP: Adenosine triphosphate
COX I: Cytochrome oxidase I
CPT-1: Carnitine acyl transferase-1
Cytc: Cytochrome C
DNA: Deoxyribonucleic acid
MAFLD: Metabolic associated fatty liver disease
MDA: Malondialdehyde
Mfn1: Mitofusin1
mPTP: Mitochondrial permeability transition pore
mtDNA: Mitochondrial deoxyribonucleic acid
NASH: Nonalcoholic steatohepatitis
NEFA: Nonesterified fatty acid
Opa1: Optic atrophy proteins
PINK1: PTEN-induced kinase 1
PPARs: Peroxisome proliferator-activated receptors
ROS: Reactive oxygen species
TG: Triglyceride
UCP-2: Mitochondrial uncoupling protein 2
VDAC1: Recombinant voltage-dependent anion channel protein 1
SIRT3: Sirtuin-3.
